# Vital pulp therapy in a 9-years-old patient: a clinical case

**DOI:** 10.1080/07853890.2021.1897414

**Published:** 2021-09-28

**Authors:** Ana Raquel Barata, Gunel Kizi, Leónia Vicente, Antonio Castaño Séiquer, Irene Ventura

**Affiliations:** a Centro de Investigação Interdisciplinar Egas Moniz (CiiEM), Egas Moniz Cooperativa de Ensino Superior, Caparica, Portugal; b Clínica Privada, Lisboa, Portugal; c Odontologia Familiar y Comunitaria, Universidad de Sevilla, Sevilla, España

## Abstract

**Introduction:**

Dental trauma is a public health problem, that have a complex aetiology, with a negative impact in the people life [1]. It has been reported that 25.7% of the children visit a dentist for the first time due to emergency situations. The most frequently reported injury seems to be damage to the enamel or the dental crowns in children [2]. Approximately 31% of dental injuries are caused by sporting activities, and many of these traumas can be prevented [3,4]. The anterosuperior teeth are, in most cases, the most injured, affecting the patient’s self-esteem. This study aims to present a clinical case of a definitive tooth that suffered a crown fracture with a pulp exposure.

**Materials and methods:**

A 9-year-old male was referred to paediatric dentistry clinic, with the chief complaint “Treat the tooth that fractured about one hour ago in the school” SIC mother. A detailed medical, dental, and social history was obtained, a clinical analysis was performed, confirming the crown trauma with a pulp exposure, positive pulpal vitality tests in the upper left incisor, without apical lesion in the radiological analysis, after signed informed consent the treatment first stage consisted in vital pulp therapy, that was performed with partial pulpotomy wherein the sealing material was MTA-Angelus^®^. In the second stage it was done a direct restoration with rubber dam technique and a silicone index using the Filtek™ Supreme XTE universal dental composite.

**Results:**

After the treatment and 2 years of follow-up the tooth presented satisfactory aesthetic and functional results and this treatment increased the patient's self-esteem. 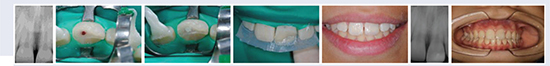

**Discussion and conclusions:**

In the present case the vital pulp therapy was performed once the tooth had complete root formation and had signs and symptoms of a pulp exposure without apical lesion. MTA has been shown to be a great pulpotomy agent its sealing ability, biocompatibility and regeneration of the original tissues when placed in contact with dental pulp tissue [5]. It is crucial to develop educational programs that emphasise the importance of prevention and the benefits of immediate treatment. Adequate diagnosis, planning and follow-up of treatment are important to get favourable results and to increase in the patient's self-esteem [6].
